# Pronucleotide Probes Reveal a Diverging Specificity for AMPylation vs UMPylation of Human and Bacterial Nucleotide Transferases

**DOI:** 10.1021/acs.biochem.3c00568

**Published:** 2024-02-22

**Authors:** Dietrich Mostert, Wilhelm Andrei Bubeneck, Theresa Rauh, Pavel Kielkowski, Aymelt Itzen, Kirsten Jung, Stephan A. Sieber

**Affiliations:** † Center for Functional Protein Assemblies (CPA), Department of Chemistry, Chair of Organic Chemistry II, Technical University of Munich, 85748 Garching, Germany; ‡ Department of Chemistry, Ludwig-Maximilians-Universität München, 81377 München, Germany; § Department of Biochemistry and Signal Transduction, University Medical Center Hamburg-Eppendorf (UKE), 20246 Hamburg, Germany; || Department of Biology I, Microbiology, Ludwig-Maximilians-Universität München, 82152 Martinsried, Germany

## Abstract

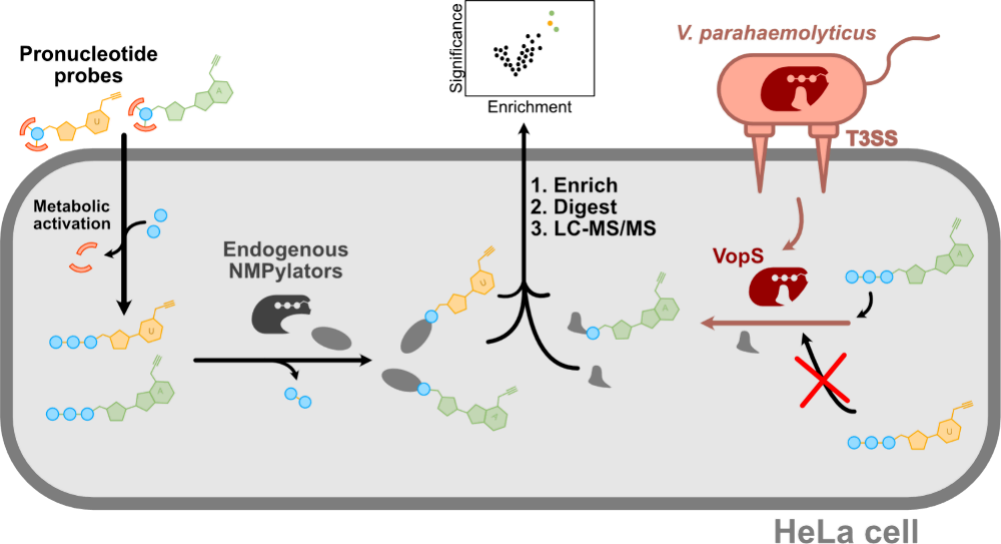

AMPylation is a post-translational modification utilized by human and bacterial cells to modulate the activity and function of specific proteins. Major AMPylators such as human FICD and bacterial VopS have been studied extensively for their substrate and target scope *in vitro*. Recently, an AMP pronucleotide probe also facilitated the *in situ* analysis of AMPylation in living cells. Based on this technology, we here introduce a novel UMP pronucleotide probe and utilize it to profile uninfected and *Vibrio parahaemolyticus* infected human cells. Mass spectrometric analysis of labeled protein targets reveals an unexpected promiscuity of human nucleotide transferases with an almost identical target set of AMP- and UMPylated proteins. Vice versa, studies in cells infected by *V. parahaemolyticus* and its effector VopS revealed solely AMPylation of host enzymes, highlighting a so far unknown specificity of this transferase for ATP. Taken together, pronucleotide probes provide an unprecedented insight into the *in situ* activity profile of crucial nucleotide transferases, which can largely differ from their *in vitro* activity.

Post-translational modifications (PTMs) largely enhance the functional scope of proteins beyond the structural diversity of the 20 natural amino acids. These modifications play crucial roles in, e.g., cellular signaling, enzyme catalysis, and the structural integrity of proteins.^1−4^ However, PTMs are not limited to enhancing the functions of proteins within a cell but can also be involved in the onset of numerous diseases.^5−8^ These include aberrant PTMs in signaling cascades leading to uncontrolled cellular growth of cancer cells^9−13^ as well as in the warfare of pathogens that dysregulate the host cell physiology.^14^ For example, bacteria have evolved numerous ways to interfere with human signaling, silencing the immune response and promoting infection.^15,16^ This interkingdom warfare is mediated by bacterial effector proteins, often transferred into human cells via type III secretion systems.^17^ Once inside the cell, these effectors mediate various PTMs, including phosphorylation, acetylation, proteolysis, and the transfer of larger molecules such as adenosine diphosphate (ADP)-ribose or adenosine monophosphate (AMP). Many effectors have common targets, such as membrane-bound guanosine triphosphatases (GTPases) from the Rho family involved in signal transduction that regulates the actin cytoskeleton and diverse immune processes as well as mitogen-activated protein kinases (MAPKs) and nuclear factor kappa B (NF-κB), which are both part of signaling pathways responsible for immune response regulation.^14,18−21^

AMPylation (also termed adenylylation) was first discovered in *Escherichia coli* as a regulatory mechanism for glutamine synthetase.^22^ In addition, several other AMPylators were discovered in bacteria. These enzymes, including VopS from *Vibrio parahaemolyticus* and IbpA from *Histophilus somni*, are secreted into host cells, where they AMPylate GTPases of the Rho family, leading to a disruption of the actin cytoskeleton and a characteristic rounded cell phenotype.^18^ The catalytic regions of both enzymes share a conserved filamentation induced by the cyclic AMP (Fic) domain mediating the covalent attachment of an AMP moiety to a Ser, Thr, or Tyr protein side chain.^19,23^ A single Fic-domain containing enzyme, termed FICD (HYPE), was also discovered in eukaryotic cells. It AMPylates the chaperone BiP (HSPA5) in the endoplasmatic reticulum, which regulates the unfolded-protein response (UPR).^4,24−26^ Moreover, the recent discovery of the pseudokinase SelO as an AMPylator in human cells highlights that Fic-independent enzymes can also catalyze this PTM.^27^

Since the discovery of AMPylation, methods to decipher the cellular substrates have been developed utilizing a diverse set of chemical probes bearing radioactive, fluorescent, or affinity reporter tags.^18,28^ Here, recent advancements in the profiling of AMPylation targets within cell lysates using ATP analogues functionalized with alkyne tags for protein enrichment via click chemistry to biotin azide, subsequent avidin enrichment, and mass spectrometric (MS) analysis revealed new potential substrates of Fic-enzymes VopS and FICD.^29,30^ We recently introduced a cell-permeable AMP pronucleotide probe (**pro-N6pA**) for identification of AMPylated proteins in intact human cells.^31,32^ In addition, this method was used to identify targets of VopS in *V. parahaemolyticus* infected human cells.^33^

While these previous efforts largely focused on the identification of AMPylation protein substrates, the transfer of alternative nucleotides, such as UMP, via these enzymes is rather underexploited. *In vitro* studies with several Fic-enzymes, including VopS and FICD, demonstrated a relaxed substrate specificity for VopS, transferring AMP, GMP, CMP, and UMP, while FICD showed efficient transfer for solely AMP.^19^ However, how these *in vitro* results translate into cellular nucleotide specificities, i.e., considering the large amount of cellular ATP as a competitor, remains elusive. Of note, studies into nucleotide specificity have focused only on the bifunctional wild-type FICD, which typically shows low AMPylation and, hence, likely low general NMPylation activity.^19^ Interestingly, a recent study with YdiU, a bacterial homologue of the human pseudokinase SelO, demonstrated the inactivation of chaperones via UMPylation, suggesting that other nucleotides could play a role in these processes.^34^

To analyze the *in situ* specificity for AMPylation vs UMPylation, we designed and synthesized a cell-permeable pronucleotide UMPylation probe (**pro-N3pU**) and applied it together with the **pro-N6pA** probe in cellular labeling studies. Here, we compared the treatment of human HeLa cells with both probes and obtained a largely similar substrate scope, highlighting that the endogenous AMPylators transfer both UMPylation and AMPylation *in situ*. On the contrary, when investigating the nucleotide transfer in human cells infected with either *V. parahaemolyticus* wild type (WT) or a VopS active site mutant strain, we obtained only protein labeling with the AMPylation probe highlighting a preference of VopS solely for this nucleotide transfer in vivo.

## Methods

### Synthesis

The synthesis of the phosphoramidate probe **pro-n3pU** is described in the Supporting Information. Chemical identity and purity of the novel probe were established using NMR and HRMS analysis.

### Cell Culture

Human epitheloid cervix carcinoma cells (HeLa) purchased from Sigma-Aldrich (93021013) were cultivated with high glucose Dulbecco’s Modified Eagle’s Medium (DMEM) supplemented with 10% fetal bovine serum (FBS) (Sigma-Aldrich) and 2 mM l-glutamine (Sigma-Aldrich) in T-175 culture flask (Sarstedt). Cells were maintained at 37 °C in a humidified 5% CO_2_ atmosphere.

### Bacterial Strains and Media

*Vibrio parahaemolyticus* strain RIMD 2210633 was received from Dr. Tetsuya Ida and Dr. Takeshi Honda from the Research Institute for Microbial Diseases, Osaka University. The bacteria were cultured in lysogeny broth (LB) medium (10 g/L casein peptone, 5 g/L NaCl, 5 g/L yeast extract, pH = 7.5) supplemented with NaCl for a total content of 3% at 30 °C, 200 r.p.m. *Vibrio parahaemolyticus* (strain RMID 2210633) mutant VopS- H348A and the VopS deletion mutant (ΔVopS) were obtained by double homologous recombination using a suicide plasmid as described in a previous study.^33^

### MTT Cytotoxicity Assay

HeLa cells were seeded at a density of 4000 cells per well in a transparent, flat-bottomed 96-well plate (200 μL medium per well). Cells were grown overnight in a humidified atmosphere at 37 °C and 5% CO_2_ to allow the cells to adhere to the surface. Subsequently, the medium was aspirated and replaced by fresh medium supplemented with **pro-N3pU** in concentrations ranging from 100 μM to 1 mM (DMSO content less than 1%) or 1% DMSO as a control. The cells were incubated at 37 °C, 5% CO_2_ for 24 h. For the determination of metabolic activity, 20 μL 3-(4,5-dimethyl-2-thiazolyl)-2,5-diphenyl-2H-tetrazolium bromide solution (MTT, 5 mg/mL in PBS) were added to each well, and the cells were incubated at 37 °C, 5% CO_2_ for 4 h. Thereafter, the medium was aspirated and the violet formazan crystals were dissolved in 200 μL DMSO per well under shaking (300 r.p.m., 10 min). Absorbance at 570 nm with a reference wavelength of 630 nm was recorded using an Infinite F200 pro plate reader (Tecan). Three biological replicates were measured for each data point. Cell viability was normalized with respect to the DMSO control (highest absorbance) and fitted by least-squares regression with a variable-slope logistic function using Prism (GraphPad). Cytotoxicity is reported as the IC50 value, the concentration at which 50% viability is reached.

### Analytical *in Situ* Labeling

HeLa cells were seeded into 6-well plates and treated with various concentrations of **pro-N3pU** for three different time periods. The previously described labeling using **pro-N6pA** at 100 μM for 16 h was included as a control. After probe treatment, the cells were harvested by carefully scraping them off and transferring them into microcentrifuge tubes. The cells were then washed with 1 mL of cold PBS. The cell pellets were reconstituted in 100 μL lysis buffer (1% NP-40, 1% sodium deoxycholate, 1 tablet protease inhibitor (cOmplete, Mini, EDTA-free protease inhibitor cocktail, Roche, 1 tablet in 15 mL). The samples were incubated for 15 min on ice and inverted twice. The lysed cells were centrifuged (21 000*g*, 5 min, 4 °C), and the soluble supernatant was transferred into a new tube. The protein concentrations of the samples were determined by BCA assay (Roti Quant, Roth), and all samples were adjusted to the same protein concentration using the lysis buffer. The samples were clicked to rhodamine-azide by copper-catalyzed azide–alkyne cycloaddition (CuAAC) with 0.2 mM rhodamine-azide, 0.1 mM TBTA ligand (1.67 mM stock in 80% *t*-BuOH, 20% DMSO, TCI), 1 mM TCEP (52 mM stock in H_2_O), and 1 mM CuSO_4_ (50 mM stock in H_2_O). The reaction was quenched by the addition of 100 μL 2× Laemmli buffer, and samples were analyzed via SDS-PAGE with fluorescent imaging.

### Preparative *in Situ* Labeling

All proteomics experiments were conducted in 4 independent biological replicates. HeLa cells were seeded into 10 cm Petri dishes and grown until 90% confluence. Cells were treated with 150 μM **pro-N3pU** or 100 μM **pro-N6pA** for 16 h (37 °C, 5% CO_2_). After probe treatment, the cells were harvested by carefully scraping them off and transferring them into Falcon tubes. The cells were then washed with 1 mL of cold PBS and transferred into microcentrifuge tubes. The cell pellets were reconstituted in 230 μL lysis buffer (1% NP-40, 1% sodium deoxycholate, 1 tablet protease inhibitor (cOmplete, Mini, EDTA-free protease inhibitor cocktail, Roche, 1 tablet in 15 mL). The samples were incubated for 15 min on ice and inverted twice. The lysed cells were centrifuged (21 000*g*, 5 min, 4 °C), and the soluble supernatant (200 μL) was transferred into a new tube. The protein concentrations of the samples were determined by BCA assay (Roti Quant, Roth), and all samples were adjusted to the same protein concentration using the lysis buffer. The samples were clicked to biotin-azide as described in the analytical labeling protocol. The reaction was quenched, and the proteins precipitated by the addition of 5-fold excess ice cold acetone and incubated overnight at −20 °C. The proteins were harvested (21 000*g*, 4 °C, 20 min) and washed twice with methanol. Therefore, the pellet was reconstituted in 500 μL methanol, sonicated (10% intensity, 10 s, Sonopuls HD 2070 ultrasonic rod, Bandelin electronic GmbH), and harvested again via centrifugation as before. Next, the proteins were reconstituted in 500 μL 0.2% SDS in PBS by sonication (10% intensity, 10 s), and the insoluble part was removed by centrifugation. The soluble fraction was added to 50 μL of washed (3× in 0.2% SDS in PBS) avidin-agarose beads and incubated for 1 h with continuous mixing. Afterward, the samples were washed three times with 0.2% SDS in PBS, two times with 6 M urea, and 3 times with PBS. For this, the samples were centrifuged for 3 min at 400*g*, and the supernatant was discarded each time. The beads were now resuspended in 200 μL digestion buffer 1 (3.6 M urea, 1.1 M thiourea, 5 mM TCEP in 20 mM HEPES, pH 7.5), and incubated for 30 min at 25 °C, 1000 rpm. The reduced bead-bound proteins were now alkylated with with 5.5 mM iodoacetamide (30 min, 1000 rpm, 25 °C), and then, the reaction was quenched with 10 mM DTT (30 min, 1000 rpm, 25 °C). Samples were first digested with 0.5 μg LysC (Wako) for 2 h at 25 °C before adding 600 μL of 50 mM TEAB with 1.5 μg trypsin (Promega) and a further incubation of 16 h at 37 °C, 1000 rpm. The digest was stopped by adding 1% FA, and the peptides were desalted using 50 mg Sep-Pak C18 cartridges (Waters Corp.). Therefore, the cartridges were equilibrated with 1 mL acetonitrile, 1 mL elution biffer (80% acetonitrile, 0.5% FA in H_2_O), and 3 mL of wash buffer 1 (0.1% TFA in H_2_O). The samples were loaded, washed with 3 mL wash buffer 1 and 0.5 mL of 0.5% FA in H_2_O. The peptides were eluted with 2 × 250 μL elution buffer and dried in a centrifugal evaporator. The peptides were reconstituted in 30 μL 1% FA and measured on an Q Exactive Plus instrument.

### Preparative *in Situ* Labeling with Infection

HeLa cells were seeded and labeled in the same way as the noninfection samples. Two additional plates were seeded which were later used to count the amount of cells per dish. In parallel, cultures of the desired *Vibrio parahaemolyticus* strains were inoculated from cryostocks and grown overnight. The overnight cultures were inoculated 1:100 into fresh medium and grown for 2.5 h. The OD_600_ of the bacterial cultures were measured, and the CFUs per μL were calculated. After counting the amount of HeLa cells on the two additional plates, the needed amount of bacteria for an MOI of 10 were harvested and then taken up in DMEM with 2 mM l-glutamine and 15 μM **pro-N3pU** or 10 μM **pro-N6pA**. The HeLa cells were washed with PBS and the respective bacteria-compound mix in DMEM was added to the plates. The cells were now incubated for 90 min at 37 °C, 5% CO_2_. The cells were harvested and further processed as described in the *in situ* labeling without infection.

### *In Vitro* UMPylation/AMPylation Assay

The *in vitro* UMPylation/AMPylation assay with recombinant VopS was performed as described previously^33^ with some minor changes. Purified VopS (AA 31–378, 1 μM) was incubated with a mix of 100 μM ATP and 100 μM UTP and the known AMPylation target Cdc42 (AA 1–188, 25 μM) in assay buffer (20 mM HEPES, pH 7.5, 100 mM NaCl, 5 mM MgCl_2_, 0.1 mg/mL BSA, 1 mM DTT) for 90 min at 30 °C. The samples were analyzed by intact protein MS (IPMS) as described previously.^33^

### Mass Spectrometry Analysis of Proteomics Samples

Peptide samples were analyzed on an UltiMate 3000 nano HPLC system (Dionex) equipped with an Acclaim C18 PepMap100 (75 μm ID × 2 cm) trap column and a 25 cm Aurora Series emitter column (25 cm × 75 μm ID, 1.6 μm FSC C18) (Ionoptics) separation column (column oven heated to 40 °C) coupled to a Q Exactive Plus Instrument (Thermo Fisher). For peptide separation, samples were loaded on the trap column and washed for 10 min with 0.1% TFA in ddH_2_O at a flow rate of 5 μL/min. Subsequently, peptides were transferred to the analytical column for peptide separation and separated using the following 132 min gradient (Buffer A: H_2_O + 0.1% FA; B: MeCN + 0.1% FA) with a flow rate of 300 nL/min: in 7 min to 5% B, in 105 min from 5 to 22%, in 10 min from 22 to 35%, and in another 10 min to 90% B. The separation gradient was followed by a column washing step using 90% B for 10 min and subsequent column re-equilibration with 5% B for 5 min. Peptides were ionized at a capillary temperature of 275 °C, and the instrument was operated in a Top12 data-dependent mode. For full scan acquisition, the Orbitrap mass analyzer was set to a resolution of *R* = 140 000, an automatic gain control (AGC) target of 3e6, and a maximal injection time of 80 ms in a scan range of 300–1500 *m*/*z*. Precursors having a charge state of >1, a minimum AGC target of 1e3, and intensities higher than 1e4 were selected for fragmentation. Peptide fragments were generated by HCD (higher-energy collisional dissociation) with a normalized collision energy of 27% and recorded in the Orbitrap at a resolution of *R* = 17 500. Moreover, the AGC target was set to 1e5 with a maximum injection time of 100 ms scan range. Dynamic exclusion duration was set to 60 s and isolation was performed in the quadrupole using a window of 1.6 *m*/*z*.

### Data Analysis

MS raw data was analyzed using MaxQuant^40^ software (version 2.0.3.0), and peptides were searched against Uniprot database for *Homo sapiens* (taxon identifier: 9606, downloaded on 14.03.2022, canonical, reviewed). For infection assays, all proteins in the UniProt database of the *Vibrio parahaemolyticus* serotype O3:K6, strain RIMD 2210633, taxon identifier: 223926, canonical version, reviewed and unreviewed proteomes, were added to the MaxQuant contaminants file. Carbamidomethylation of cysteines was set as fixed modification, and oxidation of methionines and acetylation of N-termini were set as variable modifications. Trypsin was set as proteolytic enzyme with a maximum of two missed cleavages. For main search, precursor mass tolerance was set to 4.5 ppm and fragment mass tolerance to 0.5 Da. Label free quantification (LFQ) mode was activated with an LFQ minimum ratio count of 1. A second peptide identification was enabled, and false discovery rate (FDR) determination carried out by applying a decoy database and thresholds were set to 1% FDR at peptide-spectrum match and at protein levels, and a “match between runs” (0.7 min match and 20 min alignment time windows) option was enabled. Normalized LFQ intensities extracted from the MaxQuant result table proteinGroups.txt were further analyzed with Perseus^41^ software (version 2.03.1). Prior to analysis, putative contaminants, reverse hits, and only identified by site hits were removed. Normalized LFQ intensities were log2 transformed, and proteins with at least four valid values in at least one group were used for missing value imputation from normal distribution (width 0.3, downshift 1.8, total matrix). Two-sample Students’ *t* test including Benjamini-Hochberg multiple testing correction (FDR = 0.05) was performed. The protein hits in the different various comparisons are listed in the supplementary Excel File.

## Results

### Design and Synthesis of UMPylation Pronucleotides

**Figure 1:**
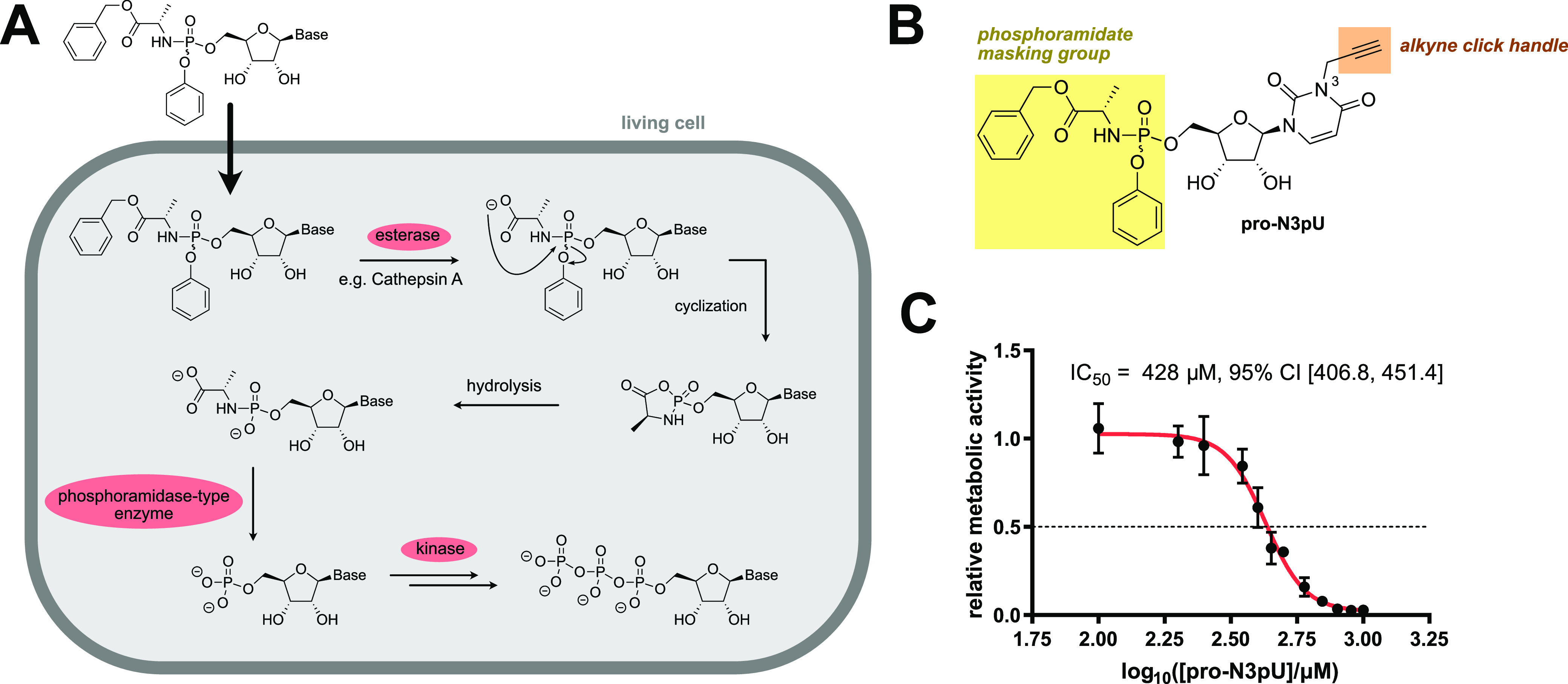
Phosphoramidate pronucelotide probes are cell-permeable and well tolerated. (A) Postulated mechanism for the metabolic activation of phosphoramidate pronucleotides in living cells.^35^ (B) Structure of the phosphoramidate UMPylation probe **pro-N3pU**. (C) MTT assay of **pro-N3pU** in HeLa cells (*n* = 3).

To study cellular UMPylation, we designed a tailored probe bearing an alkyne handle for target protein enrichment as well as a masked pronucleotide moiety for cellular uptake. The latter was already successfully applied in our first AMPylation probe generation.^31,33^ Once inside the cell, the pronucleotide is cleaved by hydrolases, and the AMP probe is subsequently converted into the triphosphate by a kinase^35^ (Figure 1A). The probe design contains two main features, the terminal alkyne handle on the heterocyclic N^3^ and the phosphoramidate prodrug moiety (Figure 1B). First, the alkyne handle is necessary for chemical proteomics in-gel and MS-based analysis. The position on the heterocyclic N^3^ possesses two advantages, the synthetic feasibility and that it likely decreases its incorporation into nucleic acids due to the hindrance of the Watson–Crick–Franklin base pairing. Second, the phosphoramidate moiety has been shown on many “highly challenging” nucleotide analogues to yield the desired modified nucleoside triphosphates.^35,36^ The structure of the UMP/CMP kinase responsible for phosphorylation of CMP and UMP analogues to the corresponding NDPs and NTPs has an induced fit active site enabling accommodation of various substrates.^37^

**Scheme 1:**
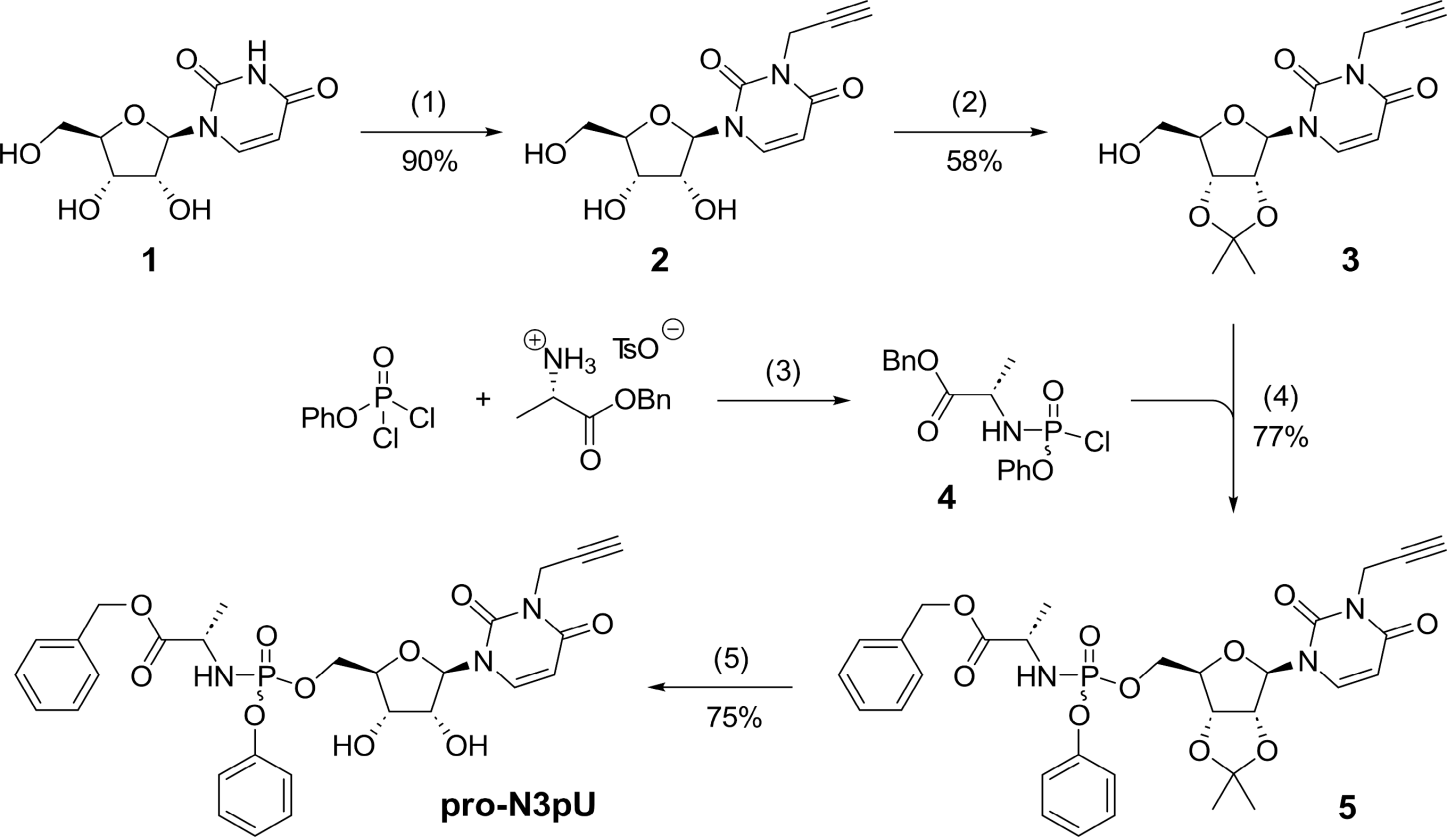
Synthesis of Uridine Phosphoramidate Probe **pro-N3pU** (1) Propargyl bromide, K_2_CO_3_, DMF, acetone, 55 °C, 2 h; (2) 2,2-dimethoxypropane, _*p*_TsOH · H_2_O, acetone, r.t., 2 h; (3) NEt_3_, CH_2_Cl_2_, −78 °C – r.t., 1 h; (4) *tert*-butyl-magnesium chloride, THF, r.t., 2 h; (5) 90% v/v TFA/H_2_O, r.t., 3 h.

The synthesis followed published procedures,^31^ starting with the alkylation of the N3-position of commercially available uridine with propargyl bromide (Scheme 1). Once the terminal alkyne handle was installed, the 2′,3′-vicinal diol was protected as an acetonide with 2,2-dimethoxypropane under acidic catalysis (58% yield). Subsequently, nucleoside derivative **3** was quantitatively deprotonated at the 5′ position using *tert*-butylmagnesium chloride and coupled with phosphochloridate **4**, affording protected phosphoramidate **5** in good yield (77%). Deprotection of the cyclic ketal using aqueous trifluoroacetic acid (TFA) afforded probe **pro-N3pU** in good yield (75%, 30% overall). The compound was isolated as a 1/1 mixture of diastereomers due to unselective substitution at the phosphorus(V) and was used as such in proteomic experiments due to the fact that the R–P and S–P isomers usually exhibit similar rates of metabolism and are difficult to separate by chromatography.^35^

### Labeling in Human Cells Reveals Promiscuity of Nucleotide Transfer

**Figure 2:**
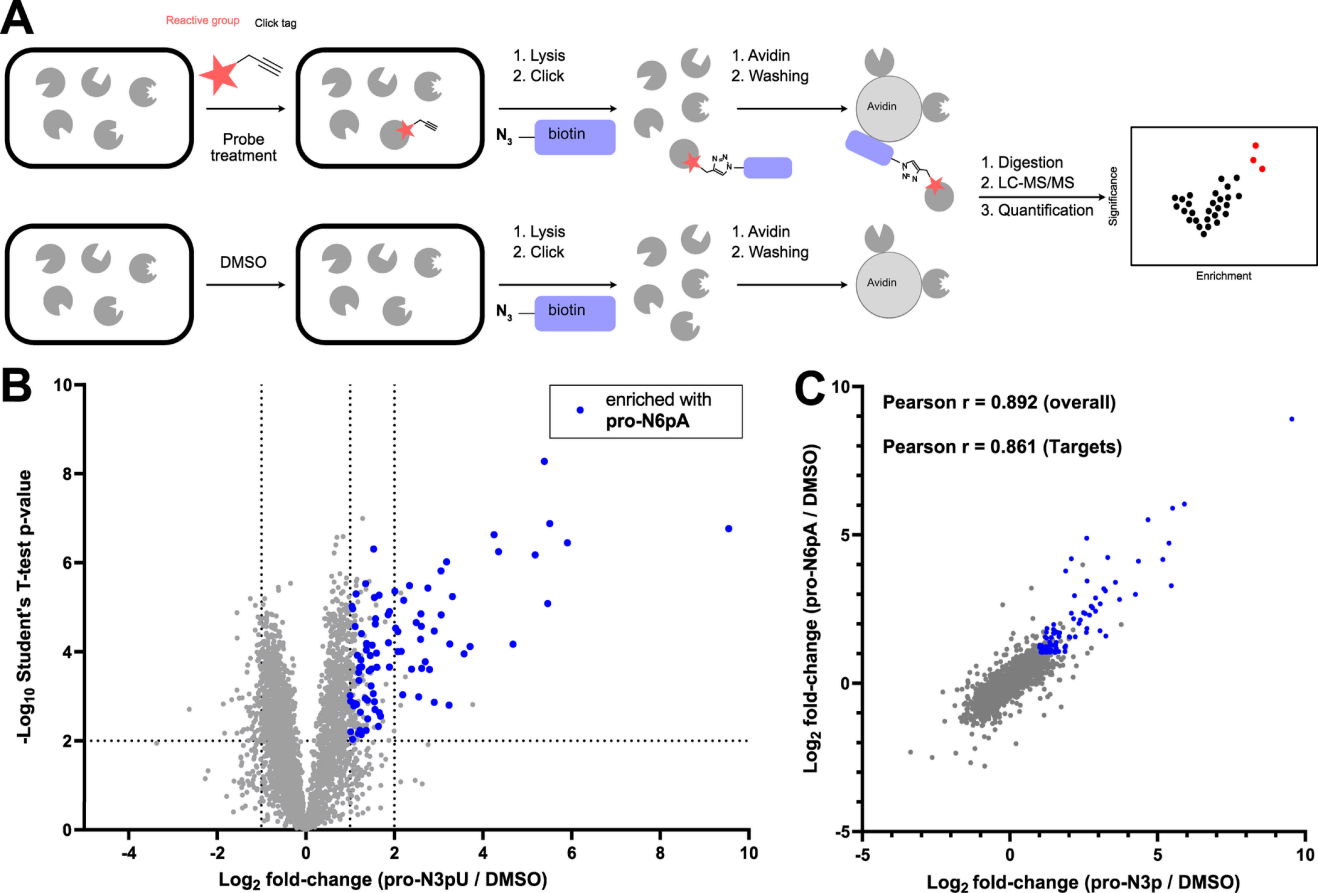
Metabolic labeling in living cells suggests a degree of promiscuity between AMPylation and UMPylation. (A) Schematic overview of the workflow for metabolic labeling using activity-based probes. (B) Volcano plot of HeLa cells treated with 150 μM **pro-N3pU** for 16 h compared to DMSO control. Proteins that are also enriched by the AMPylation probe **pro-N6pA** (*p* < 0.01 and a Log_2_(fold change) > 1) are marked in blue. Dotted lines indicate cutoff at *p* < 0.01 (*n* = 4) and a Log_2_(fold change) > 1 and Log_2_(fold change) > 2. (C) Scatter plot plotting Log_2_(fold changes) of all significant (*p* < 0.01) proteins from the **pro-N3pU** enrichment experiment against the Log_2_(fold changes) of all significant proteins from the **pro-N6pA** enrichment experiment. The overlapping protein targets from (B) are marked in blue, and the respective Pearson correlation of all proteins and of all Targets Log_2_(fold change) > 1 was calculated using prism 10.01.

Prior to **pro-N3pU** labeling in human cells, we first tested the viability of HeLa cells in the presence of the probe. Satisfyingly, toxicity was only observed at high concentrations with an IC50 value >400 μM in MTT assays (Figure 1C). The probe was subsequently incubated with intact HeLa cells for 4, 8, and 16 h at various concentrations (Figure S1A). Cell lysis followed by click chemistry to rhodamine azide and fluorescent SDS-PAGE of the labeled proteome revealed an optimal concentration of 150 μM and 16 h incubation time for clearly visible protein signals. Interestingly, a direct comparison with the **pro-N6pA** AMPylation probe resulted in an overall comparable labeling pattern (Figure S1B). In order to decipher the targets of **pro-N3pU**, we labeled HeLa cells under the optimized conditions, lysed the cells, and clicked the treated proteome with biotin azide to facilitate the enrichment of probe-modified proteins on avidin beads (Figure 2A). Tryptic digest and LC-MS/MS analysis via label-free quantification (LFQ) enables the ranking of protein hits in volcano plots compared to a DMSO control sample. Of note, both probes seem to only slightly enrich the well-known AMPylation target HSPA5 (Log_2_ fold-change of ∼0.5). The high endogenous levels of HSPA5 combined with high background binding to agarose-avidin beads could make it challenging to achieve higher enrichment values for HSPA5 with this experimental setup.

Interestingly, a side-by-side comparison of significantly (*p*-value > 0.01) enriched proteins by either **pro-N3pU** or **pro-N6pA** probes revealed a largely comparable profile of targets (Figure 2B). In fact, 37 out of 41 proteins that were enriched by **pro-N3pU** with a log2-fold enrichment >2 are also enriched by **pro-N6pA** with a log2-fold enrichment >1 among both data sets including proteins such as CTSA and CTSB, previously identified as major AMPylation targets. The overlap is still evident when comparing hits enriched by both probes by a fold-change >2 (Figure S2A). As these comparisons can be somewhat misleading depending on the fold-change cutoff used, the best way to visualize the similarities is to directly plot the fold-changes of both probes against each other and calculate the correlation. Given the vast correlation of targets by either probe (Pearson correlation *r* = 0.86) and the correlation of the fold-changes overall (Pearson correlation *r* = 0.89) (Figure 2C), we conclude that human nucleotide transferases are rather promiscuous in the substrate selection. Given the high concentration of ATP in the cell, it is remarkable that 150 μM of probe concentration was sufficient to compete for binding. Of note, this method does not distinguish between different types of AMPylating enzymes, and it is possible that more enzymes are involved in this process.

### *V. parahaemolyticus* Effector Protein VopS Solely AMPylates Human Proteins

**Figure 3:**
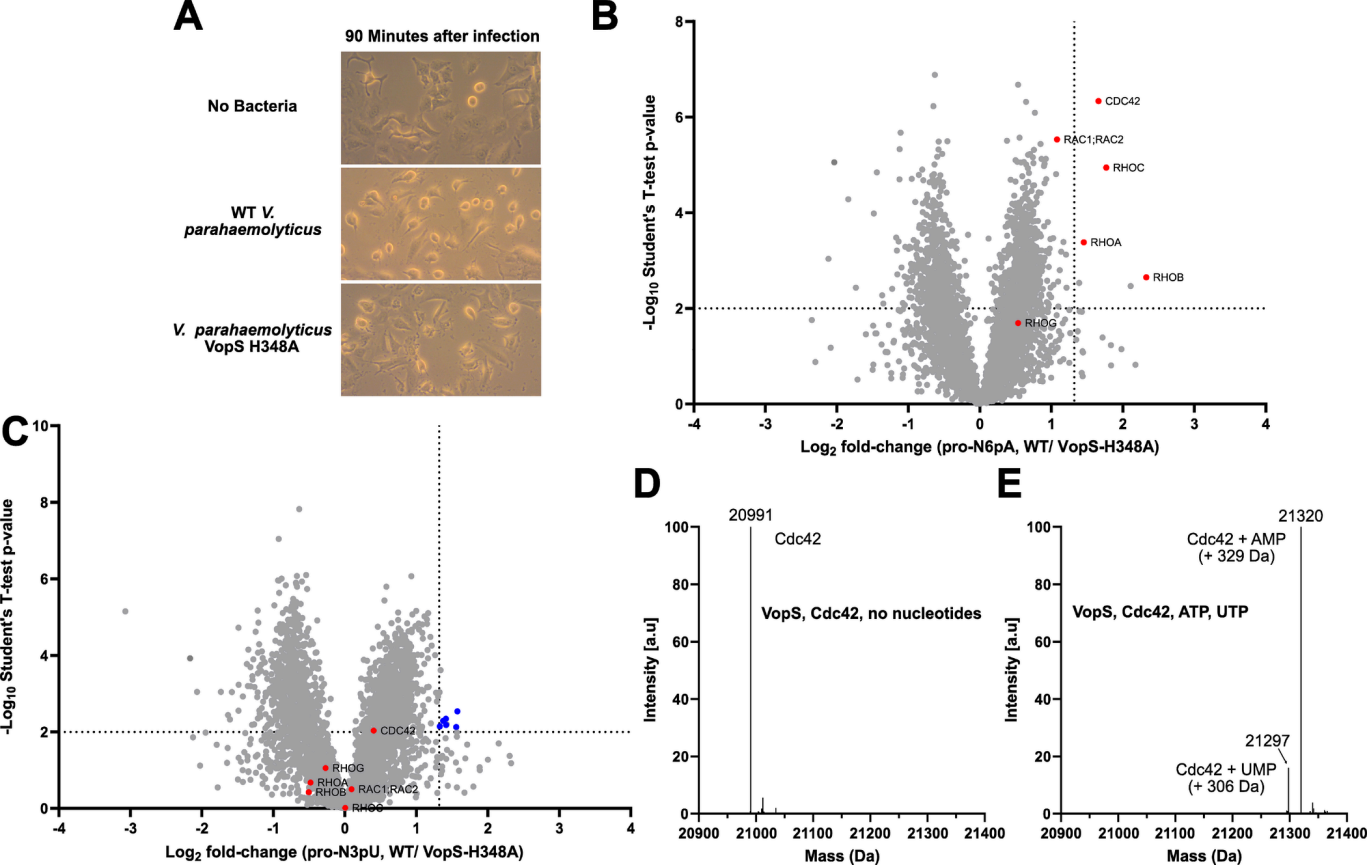
Bacterial AMPylator VopS does not UMPylate in an *in situ* infection assay. (A) Differences in phenotypic appearance when infecting HeLa cells with *V. parahaemolyticus* wild type or VopS mutant H348A for 90 min (MOI = 10). (B) Volcano plot of HeLa cells (treated with 100 μM **pro-N6pA**) infected with *V. parahaemolyticus* WT compared to VopS H348 mutant infection. Dotted lines indicate cutoff at *p* < 0.01 (*n* = 4) and a fold change >2.5 (Log_2_ > 1.322). Known VopS AMPylation targets are highlighted in red. (C) Volcano plot of HeLa cells (treated with 150 μM **pro-N3pU**) infected with *V. parahaemolyticus* WT compared to VopS H348 mutant infection. Dotted lines indicate cutoff at *p* < 0.01 (*n* = 4) and a fold change >2.5 (Log_2_ > 1.322). Known VopS AMPylation targets are highlighted in red. False positive hits are marked in blue, and their respective profile plots are shown in Figure S3D. (D) No nucleotide control of *in vitro* assay of VopS and the substrate Cdc42. Intact protein mass of Cdc42 (monoisotopic) without any modification. Figure is representative of three independent replicates. (E) *In vitro* assay of VopS and the substrate Cdc42 with 100 μM ATP and UTP (equimolar). Intact protein mass of Cdc42 (monoisotopic) modified with AMP and, to a far lesser extent, UMP. Figure is representative of three independent replicates.

Intrigued by the relaxed substrate tolerance of human AMPylators, we turned our attention to the class of protein nucleotide transferases in bacteria. VopS of *V. parahaemolyticus* was previously shown to address a specific set of rho GTPases in human cells via the **pro-N6pA** probe.^33^ To investigate if the reported *in vitro* substrate tolerance of VopS toward different nucleotide substrates^19^ also holds true for its action in living cells during infection, we applied our novel **pro-N3pU** probe in HeLa cells infected with *V. parahaemolyticus*. Cells were infected with bacteria in a multiplicity of infection (MOI) of 1:10, resulting in characteristic round-shaped cells after 90 min (Figure 3A). Accordingly, a strain carrying the corresponding VopS active site H348A mutation did not affect HeLa cell morphology.

With these established conditions, we pretreated HeLa cells individually with both **pro-N6pA** and **pro-N3pU** cells prior to the infection with bacteria. Cells were prepared as described above, and LC-MS/MS based proteome analysis revealed a comparable **pro-N6pA** enrichment of target proteins as observed before^33^ that was absent in the mutant control (Figure 3B). Strikingly, no significant protein enrichment was observed with **pro-N3pU**, highlighting that VopS performs AMPylation but no UMPylation of human protein targets (Figures 3C, S3A). Both probes exhibit the same enrichment pattern of endogenously enriched samples in the infection assay, but while **pro-N6pA** additionally enriches the known VopS substrates (Figure S3B), the UMPylation probe **pro-N3pU** only enriches the endogenously AMP/UMPylated targets and not any of the VopS targets (Figure S3C), highlighting the diverging specificity of VopS and the human AMPylators. The intensity of the known VopS targets across the differently infected samples (Figure S3D) further shows that only **pro-N6pA** treated cells infected with wild type *V. parahaemolyticus* enrich these targets, while false positives from the **pro-N3pU** experiment (blue lines) arise from missing value imputation in the mutant treated samples and not enrichment by the wild type samples. To rule out that the diverging nucleotide promiscuity of human AMPylators and VopS is only due to the shorter time span (16 h vs 90 min), we labeled HeLa cells with both probes for only 90 min (Figure S2B,C). As expected, the overall enrichment is significantly lower for both probes. However, both probes lead to similar labeling patterns, also after only 90 min. This confirms that the human AMPylators are indeed more promiscuous than the bacterial VopS, even when given the same amount of time for labeling. To finally validate this substrate specificity, we performed an *in vitro* experiment with recombinant VopS and Cdc42 as a cognate substrate. Enzymes were incubated with 100 μM ATP and 100 μM UTP for 90 min at 30 °C before being analyzed by intact protein mass spectrometry (IP-MS) (Figure 3D,E). For this experiment, we assume that the ionization potential of Cdc42-AMP and Cdc42-UMP are the same. In intact protein MS, the ionization potential is mainly driven by the amino acid sequence. Both AMP and UMP differ only slightly in size but introduce the same charge to the protein and, therefore, should not differ in their effect on the ionization potential. When VopS and Cdc42 are incubated with an equimolar amount of ATP and UTP, the preferred substrate is clearly ATP. While both Cdc42-adducts (+AMP, +UMP) are detected, the signal for Cdc42-AMP is far greater than the signal for Cdc42-UMP (Figure 3E). When taking into account the higher intracellular concentration of ATP (3152 μM) compared to UTP (576 μM),^38^ this *in vitro* preference for AMPylation translates to a negligible rate of UMPylation *in vivo*, as seen in the metabolic labeling experiments using the pronucleotide probes.

## Conclusion

We here showcase the utility of our pronucleotide probes to decipher the substrate and target scope of human and bacterial nucleotide transferases and demonstrate the need for complementary approaches in the study of enzymes under *in vitro* and *in situ* conditions. While assays with the recombinant VopS enzyme indicated a relatively relaxed specificity for several nucleotides,^19^ our *in situ* labeling suggests at least no tolerance for UTP as an alternative substrate to ATP. Vice versa, human nucleotide transferases exhibited a rather relaxed substrate tolerance with our probes, which was unexpected given the previously obtained specificity of FICD solely for ATP *in vitro*.^19^ However, we cannot exclude that other nucleotide transferases, such as SelO, modify protein targets more promiscuously. Future studies into the distinct targets of different human AMPylators and their potential difference in nucleotide promiscuity could give further insights into the role of different NMPylations in human cells. For example, AMPylation was shown to play an important role during neuronal development and degeneration.^39^ Understanding how this substrate promiscuity might translate into different phenotypes under varying cellular conditions could give further insights into the role of these PTMs during these processes. To gain further insight into the substrate promiscuity of different human NMPylators, the pronucleotide probes represent an ideal tool. The probes could be used in various knock-down cells to identify the substrate scope and the nucleotide specificity of different known and putative NMPylators. Acquiring such a detailed understanding of different NMPylators would be the foundation for targeting this enzyme class for clinical use. We thus conclude that our *in situ* pronucleotides are versatile tools in the study of PTMs in living cells, reporting target proteins as well as substrate tolerance of transferases in different organisms.

## Data Availability

The mass spectrometry proteomics data have been deposited to the ProteomeXchange Consortium via the PRIDE^42^ partner repository with the data set identifier PXD045925

## Supporting Information Available

Figure S1: Initial analytical labeling using **pro-N3pU** to determine ideal conditions visualized by in-gel fluorescence after clicking treated samples with rhodamine azide. Figure S2: Comparison of endogenous UMP and AMP targets in HeLa cells represented in a Venn diagram and volcano plots of HeLa cells treated with both probes for only 90 min. Figure S3: Further volcano plots of HeLa cells (treated with the pronucleotide probes) infected with *V. parahaemolyticus* WT compared to no bacterial infection. Supplementary methods with detailed synthesis of **pro-N3pU** (PDF)
Supplementary table of all protein hits in various experiments (XLSX)

## Accession Codes

VopS: Q87P32 Cdc42: P60953

## References

[ref1] Walsh C. T.; Garneau-Tsodikova S.; Gatto G. J. Protein Posttranslational Modifications: The Chemistry of Proteome Diversifications. Angewandte Chemie - International Edition 2005, 44, 7342–7372. 10.1002/anie.200501023. 16267872

[ref2] Aebersold R.; Agar J. N.; Amster I. J.; Baker M. S.; Bertozzi C. R.; Boja E. S.; Costello C. E.; Cravatt B. F.; Fenselau C.; Garcia B. A.; Ge Y.; Gunawardena J.; Hendrickson R. C.; Hergenrother P. J.; Huber C. G.; Ivanov A. R.; Jensen O. N.; Jewett M. C.; Kelleher N. L.; Kiessling L. L.; Krogan N. J.; Larsen M. R.; Loo J. A.; Ogorzalek Loo R. R.; Lundberg E.; Maccoss M. J.; Mallick P.; Mootha V. K.; Mrksich M.; Muir T. W.; Patrie S. M.; Pesavento J. J.; Pitteri S. J.; Rodriguez H.; Saghatelian A.; Sandoval W.; Schlüter H.; Sechi S.; Slavoff S. A.; Smith L. M.; Snyder M. P.; Thomas P. M.; Uhlén M.; Van Eyk J. E.; Vidal M.; Walt D. R.; White F. M.; Williams E. R.; Wohlschlager T.; Wysocki V. H.; Yates N. A.; Young N. L.; Zhang B. How Many Human Proteoforms Are There?. Nat. Chem. Biol. 2018, 14 (3), 206–214. 10.1038/nchembio.2576. 29443976 PMC5837046

[ref3] Casey A. K.; Orth K. Enzymes Involved in AMPylation and DeAMPylation. Chem. Rev. 2018, 118 (3), 1199–1215. 10.1021/acs.chemrev.7b00145. 28819965 PMC5896785

[ref4] Ham H.; Woolery A. R.; Tracy C.; Stenesen D.; Krämer H.; Orth K. Unfolded Protein Response-Regulated Drosophila Fic (DFic) Protein Reversibly AMPylates BiP Chaperone during Endoplasmic Reticulum Homeostasis. J. Biol. Chem. 2014, 289 (52), 36059–36069. 10.1074/jbc.M114.612515. 25395623 PMC4276871

[ref5] Vogl A. M.; Brockmann M. M.; Giusti S. A.; Maccarrone G.; Vercelli C. A.; Bauder C. A.; Richter J. S.; Roselli F.; Hafner A.-S.; Dedic N.; Wotjak C. T.; Vogt-Weisenhorn D. M.; Choquet D.; Turck C. W.; Stein V.; Deussing J. M.; Refojo D. Neddylation Inhibition Impairs Spine Development, Destabilizes Synapses and Deteriorates Cognition. Nat. Neurosci. 2015, 18 (2), 239 10.1038/nn.3912. 25581363

[ref6] Song Y.; Brady S. T. Post-Translational Modifications of Tubulin: Pathways to Functional Diversity of Microtubules. Trends Cell Biol. 2015, 25 (3), 125–136. 10.1016/j.tcb.2014.10.004. 25468068 PMC4344850

[ref7] Magiera M. M.; Singh P.; Gadadhar S.; Janke C. Tubulin Posttranslational Modifications and Emerging Links to Human Disease. Cell 2018, 173 (6), 1323–1327. 10.1016/j.cell.2018.05.018. 29856952

[ref8] Rebelo A. P.; Ruiz A.; Dohrn M. F.; Wayand M.; Farooq A.; Danzi M. C.; Beijer D.; Aaron B.; Vandrovcova J.; Houlden H.; Matalonga L.; Abreu L.; Rouleau G.; Estiar M. A.; Van de Vondel L.; Gan-Or Z.; Baets J.; Schüle R.; Zuchner S. BiP Inactivation Due to Loss of the DeAMPylation Function of FICD Causes a Motor Neuron Disease. Genet. Med. 2022, 24 (12), 2487–2500. 10.1016/j.gim.2022.08.019. 36136088

[ref9] Anbalagan M.; Huderson B.; Murphy L.; Rowan B. G. Post-Translational Modifications of Nuclear Receptors and Human Disease. Nucl. Recept. Signal. 2012, 10, nrs.10001 10.1621/nrs.10001. PMC3309075 22438791

[ref10] Narayan S.; Bader G. D.; Reimand J. Frequent Mutations in Acetylation and Ubiquitination Sites Suggest Novel Driver Mechanisms of Cancer. Genome Med. 2016, 8 (1), 1–13. 10.1186/s13073-016-0311-2. 27175787 PMC4864925

[ref11] Wang Y.; Zhang J.; Li B.; He Q. Y. Advances of Proteomics in Novel PTM Discovery: Applications in Cancer Therapy. Small Methods. 2019, 3 (5), 1900041 10.1002/smtd.201900041.

[ref12] Holstein E.; Dittmann A.; Kääriäinen A.; Pesola V.; Koivunen J.; Pihlajaniemi T.; Naba A.; Izzi V. The Burden of Post-translational Modification (Ptm)— Disrupting Mutations in the Tumor Matrisome. Cancers (Basel) 2021, 13 (5), 1081 10.3390/cancers13051081. 33802493 PMC7959462

[ref13] Tikhonov D.; Kulikova L.; Kopylov A. T.; Rudnev V.; Stepanov A.; Malsagova K.; Izotov A.; Kulikov D.; Zulkarnaev A.; Enikeev D.; Potoldykova N.; Kaysheva A. L. Proteomic and Molecular Dynamic Investigations of PTM-Induced Structural Fluctuations in Breast and Ovarian Cancer. Sci. Rep. 2021, 11 (1), 19318 10.1038/s41598-021-98201-7. 34588485 PMC8481388

[ref14] Ham H.; Sreelatha A.; Orth K. Manipulation of Host Membranes by Bacterial Effectors. Nat. Publ. Gr. 2011, 9, 635 10.1038/nrmicro2602. 21765451

[ref15] Li H.; Xu H.; Zhou Y.; Zhang J.; Long C.; Li S.; Chen S.; Zhou J. M.; Shao F. The Phosphothreonine Lyase Activity of a Bacterial Type III Effector Family. Science (80-.) 2007, 315 (5814), 1000–1003. 10.1126/science.1138960. 17303758

[ref16] Arbibe L.; Kim D. W.; Batsche E.; Pedron T.; Mateescu B.; Muchardt C.; Parsot C.; Sansonetti P. J. An Injected Bacterial Effector Targets Chromatin Access for Transcription Factor NF-JB to Alter Transcription of Host Genes Involved in Immune Responses. Nat Immunol 2007, 8, 47 10.1038/ni1423. 17159983

[ref17] Hauser A. R. The Type III Secretion System of Pseudomonas Aeruginosa: Infection by Injection. Nature Reviews Microbiology 2009, 7, 654–665. 10.1038/nrmicro2199. 19680249 PMC2766515

[ref18] Yarbrough M. L.; Li Y.; Kinch L. N.; Grishin N. V.; Ball H. L.; Orth K. AMPylation of Rho GTPases by Vibrio VopS Disrupts Effector Binding and Downstream Signaling. Science (80-.) 2009, 323 (5911), 269–272. 10.1126/science.1166382. 19039103

[ref19] Mattoo S.; Durrant E.; Chen M. J.; Xiao J.; Lazar C. S.; Manning G.; Dixon J. E.; Worby C. A. Comparative Analysis of Histophilus Somni Immunoglobulin-Binding Protein A (IbpA) with Other Fic Domain-Containing Enzymes Reveals Differences in Substrate and Nucleotide Specificities. J. Biol. Chem. 2011, 286 (37), 32834–32842. 10.1074/jbc.M111.227603. 21795713 PMC3173180

[ref20] Ribet D.; Cossart P. Post-Translational Modifications in Host Cells during Bacterial Infection. FEBS Lett. 2010, 584, 2748–2758. 10.1016/j.febslet.2010.05.012. 20493189

[ref21] Müller M. P.; Peters H.; Blümer J.; Blankenfeldt W.; Goody R. S.; Itzen A. The Legionella Effector Protein DrrA AMPylates the Membrane Traffic Regulator Rab1b. Science (80-.) 2010, 329 (5994), 946–949. 10.1126/science.1192276. 20651120

[ref22] Jiang P.; Peliska J. A.; Ninfa A. J. The Regulation of Escherichia Coli Glutamine Synthetase Revisited: Role of 2-Ketoglutarate in the Regulation of Glutamine Synthetase Adenylylation State. Biochemistry 1998, 37 (37), 12802–12810. 10.1021/bi980666u. 9737857

[ref23] Engel P.; Goepfert A.; Stanger F. V.; Harms A.; Schmidt A.; Schirmer T.; Dehio C. Adenylylation Control by Intra- or Intermolecular Active-Site Obstruction in Fic Proteins. Nature 2012, 482, 107 10.1038/nature10729. 22266942

[ref24] Sanyal A.; Chen A. J.; Nakayasu E. S.; Lazar C. S.; Zbornik E. A.; Worby C. A.; Koller A.; Mattoo S. A Novel Link between Fic (Filamentation Induced by CAMP)-Mediated Adenylylation/AMPylation and the Unfolded Protein Response. Journal of Biological Chemistry 2015, 290, 8482 10.1074/jbc.M114.618348. 25601083 PMC4375499

[ref25] Preissler S.; Rato C.; Chen R.; Antrobus R.; Ding S.; Fearnley I. M.; Ron D. AMPylation Matches BiP Activity to Client Protein Load in the Endoplasmic Reticulum. Elife 2015, 4, e12621 10.7554/eLife.12621. 26673894 PMC4739761

[ref26] Preissler S.; Rato C.; Perera L. A.; Saudek V.; Ron D. FICD Acts Bifunctionally to AMPylate and De-AMPylate the Endoplasmic Reticulum Chaperone BiP. Nat. Publ. Gr. 2017, 24, 23 10.1038/nsmb.3337. PMC5221731 27918543

[ref27] Sreelatha A.; Yee S. S.; Lopez V. A.; Park B. C.; Kinch L. N.; Pilch S.; Servage K. A.; Zhang J.; Jiou J.; Karasiewicz-Urbańska M.; Łobocka M.; Grishin N. V.; Orth K.; Kucharczyk R.; Pawłowski K.; Tomchick D. R.; Tagliabracci V. S. Protein AMPylation by an Evolutionarily Conserved Pseudokinase. Cell 2018, 175 (3), 809–821.e19. 10.1016/j.cell.2018.08.046. 30270044 PMC6524645

[ref28] Hao Y. H.; Chuang T.; Ball H. L.; Luong P.; Li Y.; Flores-Saaib R. D.; Orth K. Characterization of a Rabbit Polyclonal Antibody against Threonine-AMPylation. J. Biotechnol. 2011, 151 (3), 251–254. 10.1016/j.jbiotec.2010.12.013. 21185336 PMC4391625

[ref29] Grammel M.; Luong P.; Orth K.; Hang H. C. A Chemical Reporter for Protein AMPylation. J. Am. Chem. Soc. 2011, 133 (43), 17103–17105. 10.1021/ja205137d. 21942216 PMC3246509

[ref30] Gulen B.; Rosselin M.; Fauser J.; Albers M. F.; Pett C.; Krisp C.; Pogenberg V.; Schlüter H.; Hedberg C.; Itzen A. Identification of Targets of AMPylating Fic Enzymes by Co-Substrate-Mediated Covalent Capture. Nat. Chem. 2020, 12 (8), 732–739. 10.1038/s41557-020-0484-6. 32632184

[ref31] Kielkowski P.; Buchsbaum I. Y.; Kirsch V. C.; Bach N. C.; Drukker M.; Cappello S.; Sieber S. A. FICD Activity and AMPylation Remodelling Modulate Human Neurogenesis. Nat. Commun. 2020, 11 (1), 1–13. 10.1038/s41467-019-14235-6. 31980631 PMC6981130

[ref32] Kielkowski P.; Buchsbaum I. Y.; Becker T.; Bach K.; Cappello S.; Sieber S. A. A Pronucleotide Probe for Live-Cell Imaging of Protein AMPylation. ChemBioChem. 2020, 21 (9), 1285–1287. 10.1002/cbic.201900716. 32027064 PMC7317759

[ref33] Rauh T.; Brameyer S.; Kielkowski P.; Jung K.; Sieber S. A. MS-Based in Situ Proteomics Reveals AMPylation of Host Proteins during Bacterial Infection. ACS Infect. Dis. 2020, 6 (12), 3277–3289. 10.1021/acsinfecdis.0c00740. 33259205 PMC9558369

[ref34] Yang Y.; Yue Y.; Song N.; Li C.; Yuan Z.; Wang Y.; Ma Y.; Li H.; Zhang F.; Wang W.; Jia H.; Li P.; Li X.; Wang Q.; Ding Z.; Dong H.; Gu L.; Li B. The YdiU Domain Modulates Bacterial Stress Signaling through Mn2+-Dependent UMPylation. Cell Rep. 2020, 32 (12), 108161 10.1016/j.celrep.2020.108161. 32966796

[ref35] Mehellou Y.; Rattan H. S.; Balzarini J. The ProTide Prodrug Technology: From the Concept to the Clinic. J. Med. Chem. 2018, 61 (6), 2211–2226. 10.1021/acs.jmedchem.7b00734. 28792763 PMC7075648

[ref36] Pradere U.; Garnier-Amblard E. C.; Coats S. J.; Amblard F.; Schinazi R. F. Synthesis of Nucleoside Phosphate and Phosphonate Prodrugs. Chem. Rev. 2014, 114 (18), 9154–9218. 10.1021/cr5002035. 25144792 PMC4173794

[ref37] Segura-Peña D.; Sekulic N.; Ort S.; Konrad M.; Lavie A. Substrate-Induced Conformational Changes in Human UMP/CMP Kinase. Journal of Biological Chemistry 2004, 279, 33882 10.1074/jbc.M401989200. 15163660

[ref38] Traut T. W. Physiological Concentrations of Purines and Pyrimidines. Mol. Cell. Biochem. 1994, 140 (1), 1–22. 10.1007/BF00928361. 7877593

[ref39] Sieber S. A.; Cappello S.; Kielkowski P. From Young to Old: AMPylation Hits the Brain. Cell Chem. Biol. 2020, 27 (7), 773–779. 10.1016/j.chembiol.2020.05.009. 32521229

[ref40] Cox J.; Mann M. MaxQuant Enables High Peptide Identification Rates, Individualized p.p.b.-Range Mass Accuracies and Proteome-Wide Protein Quantification. Nat. Biotechnol. 2008, 26 (12), 1367–1372. 10.1038/nbt.1511. 19029910

[ref41] Tyanova S.; Temu T.; Sinitcyn P.; Carlson A.; Hein M. Y.; Geiger T.; Mann M.; Cox J. The Perseus Computational Platform for Comprehensive Analysis of (Prote)Omics Data. Nat. Methods 2016, 13 (9), 731–740. 10.1038/nmeth.3901. 27348712

[ref42] Perez-Riverol Y.; Bai J.; Bandla C.; García-Seisdedos D.; Hewapathirana S.; Kamatchinathan S.; Kundu D. J.; Prakash A.; Frericks-Zipper A.; Eisenacher M.; Walzer M.; Wang S.; Brazma A.; Vizcaíno J. A. The PRIDE Database Resources in 2022: A Hub for Mass Spectrometry-Based Proteomics Evidences. Nucleic Acids Res. 2022, 50, D543 10.1093/nar/gkab1038. 34723319 PMC8728295

