# Supplementary material for: Pronucleotide Probes Reveal a Diverging Specificity for AMPylation vs UMPylation of Human and Bacterial Nucleotide Transferases
Source: Biochemistry. 2024 Feb 22;63(5):651–9. doi: 10.1021/acs.biochem.3c00568 (PMC10918828; doi:10.1021/acs.biochem.3c00568)
Supplement: Supplementary file 1 — bi3c00568_si_001.pdf [file bi3c00568_si_001.pdf]

Pronucleotide probes reveal a diverging specificity for AMPylation vs. UMPylation of human and bacterial nucleotide transferases

Dietrich Mostert[†], Wilhelm Andrei Bubeneck[†], Theresa Rauh[†], Pavel Kielkowski[‡], Aymelt Itzen[§], Kirsten Jung[||] and Stephan A. Sieber[†]*

[†]: Center for Functional Protein Assemblies (CPA), Department of Chemistry, Chair of Organic Chemistry II, Technical University of Munich, 85748 Garching, Germany

[‡]: Department of Chemistry, Ludwig-Maximilians-Universität München, 81377 München, Germany

[§]: Department of Biochemistry and Signal Transduction, University Medical Center Hamburg-Eppendorf (UKE), 20246, Hamburg, Germany

[||]: Department of Biology I, Microbiology, Ludwig-Maximilians-Universität München, 82152 Martinsried, Germany

*: Corresponding author; E-mail: stephan.sieber@tum.de

Supporting Information

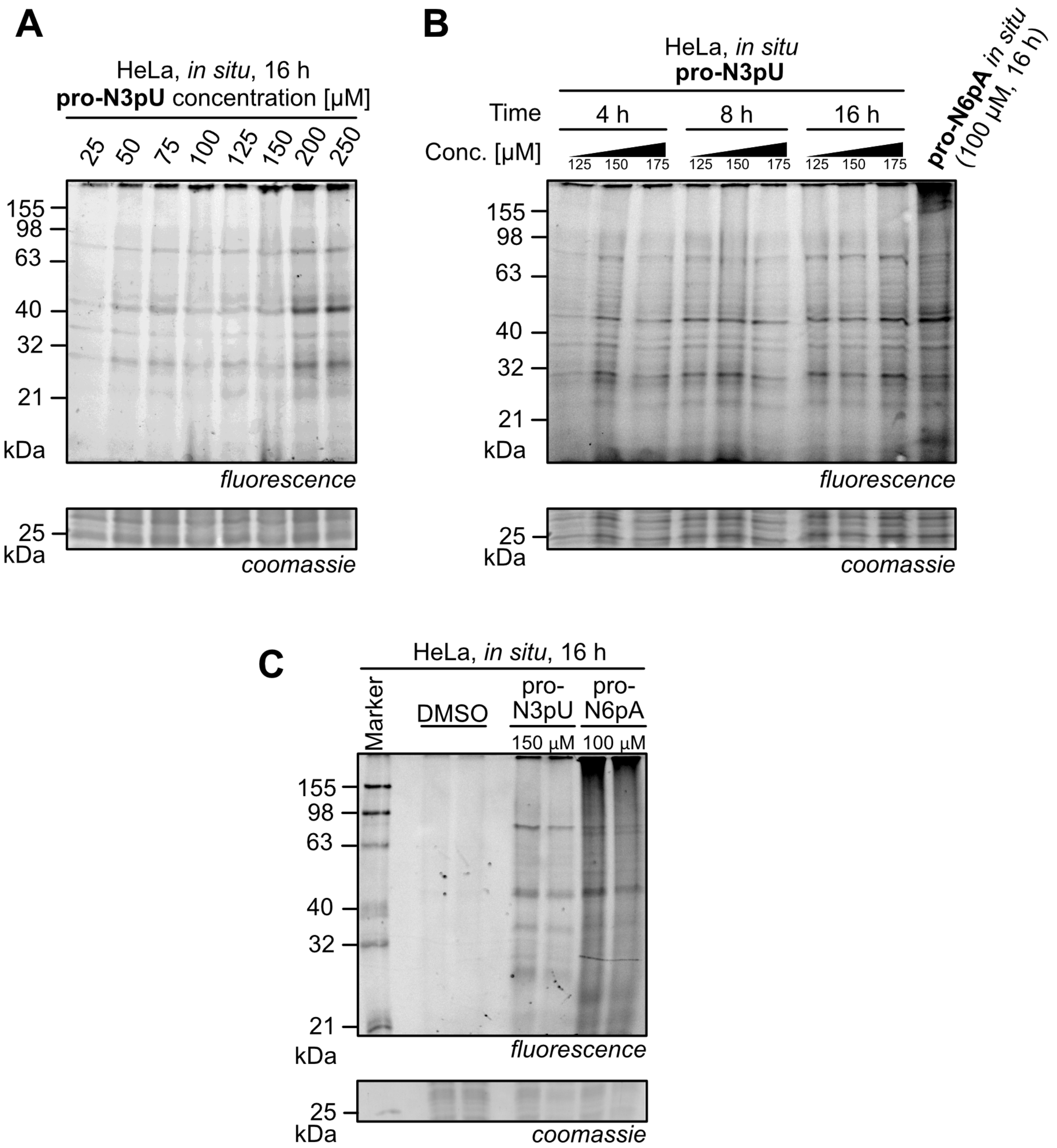

Figure S1. Initial analytical labeling using **pro-N3pU** to determine ideal conditions visualized by in-gel fluorescence after clicking treated samples with rhodamine azide. (A) Concentration-dependent labeling (B) Concentration- and time-dependent labeling, including a direct comparison with AMPylation probe **pro-N6pA**. (C) Analytical labeling in human cells, including a DMSO control to exclude unspecific fluorescence (Duplicates).

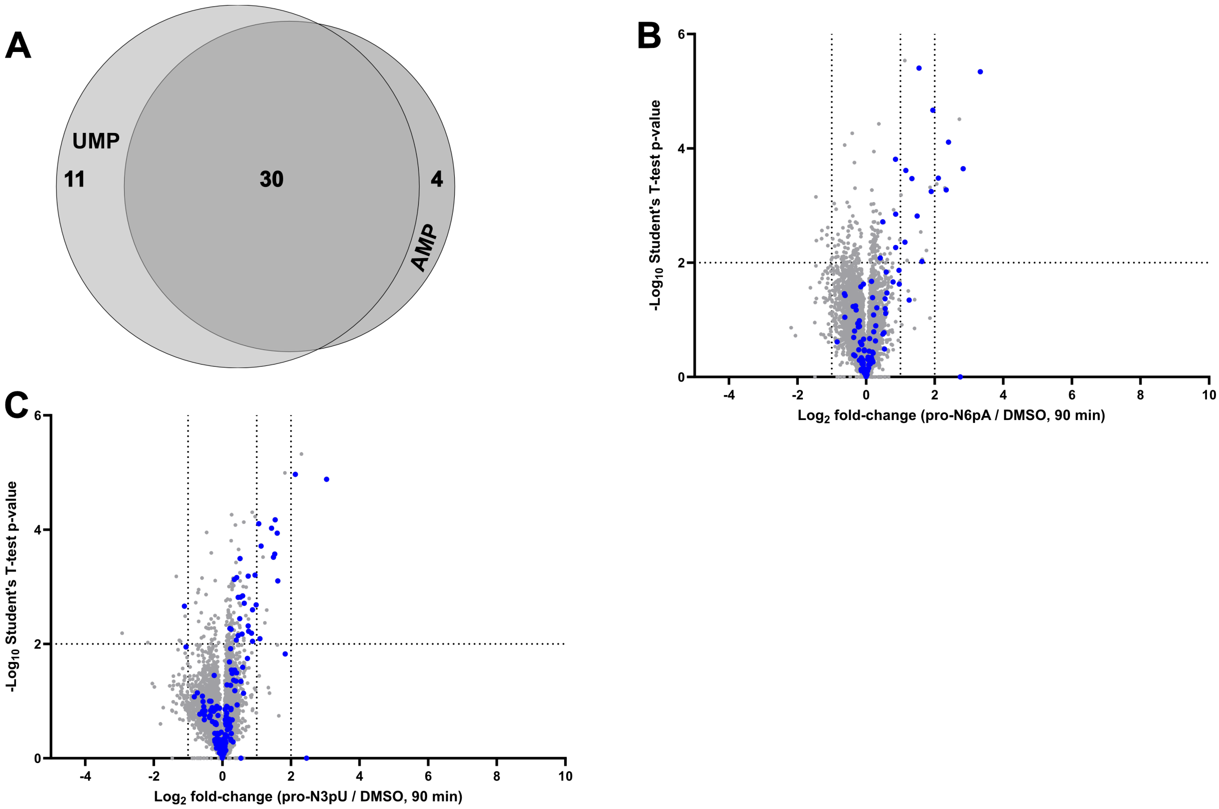

Figure S2. (A) Venn-Diagram of all proteins enriched by the **pro-N3pU** and **pro-N6pA** probes by a fold-change of more than 2. There is a significant overlap of both probes. Of note, some of the proteins that do not overlap are still enriched by both probes, just not to the same extent. (B, C) Volcano-plot of HeLa cells treated with 150 µM **pro-N6pA** (B) or 100 µM **pro-N3pU** (C) for 90 minutes compared to DMSO control. Proteins that are also enriched by the respective probe after 16h h incubation ($p < 0.01$ and a Log_2(fold change) > 1) are marked in blue. Dotted lines indicate cut-off at $p < 0.01$ (n=4) and a Log_2(fold change) > 1 and Log_2(fold change) > 2. Both probes enrich many of the same proteins as after 16 h incubation, albeit to a lesser extent.

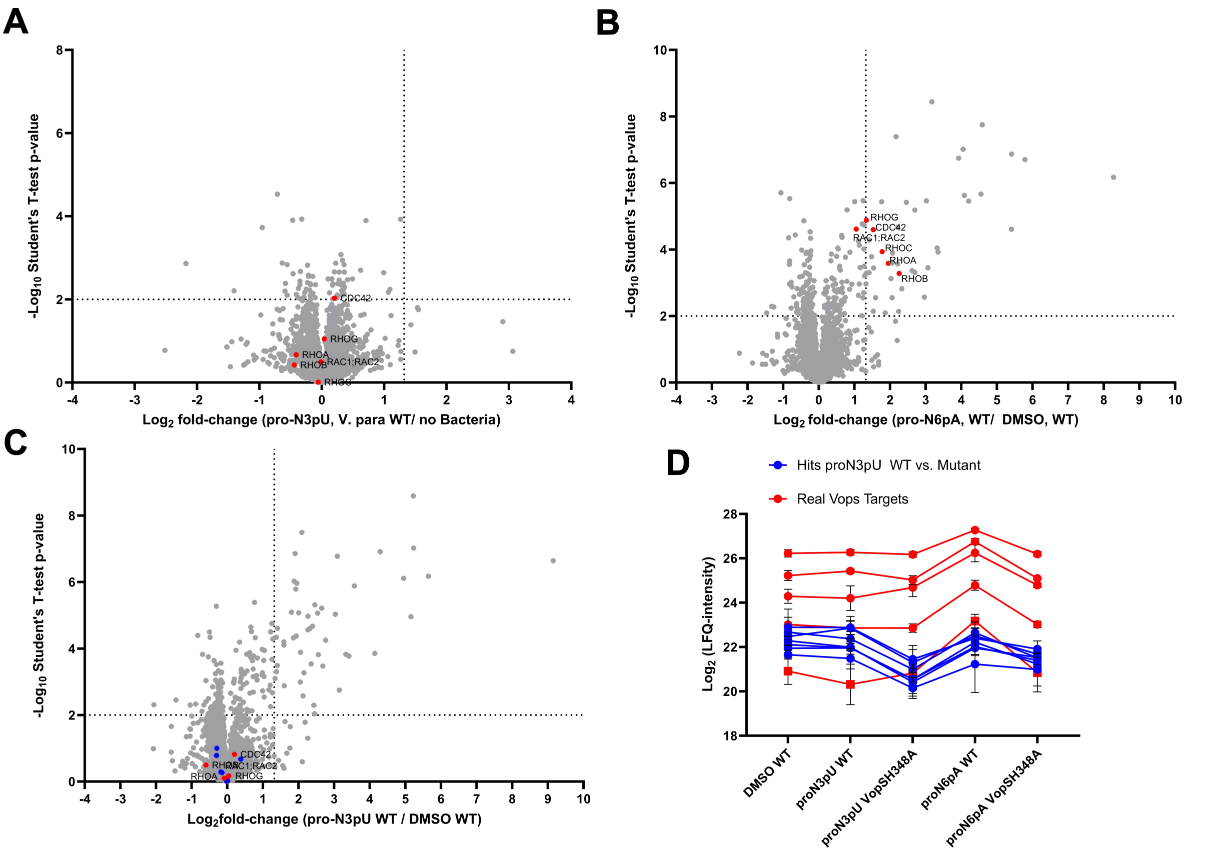

Figure S3. (A) Volcano plot of HeLa cells (treated with 150 μM **pro-N3pU**) infected with *V. parahaemolyticus* WT compared to no bacterial infection; (B) HeLa cells (treated with 100 μM **pro-N6pA**) infected with *V. parahaemolyticus* WT compared to HeLa cells (treated with DMSO) infected with *V. parahaemolyticus* WT; (C) HeLa cells (treated with 150 μM **pro-N3pU**) infected with *V. parahaemolyticus* WT compared to HeLa cells (treated with DMSO) infected with *V. parahaemolyticus* WT. Dotted lines indicate cut-off at $p < 0.01$ (n=4) and a fold change > 2.5 ($Log_2 > 1.322$). Known VopS AMPylation targets are highlighted in red. False positive hits from Figure 3C are marked in blue and their respective profile plots are shown in Figure S3D. (D) Profile plots of the VopS targets and apparently enriched proteins from Figure 3C. False hits (blue) are not enriched by the probe but are merely less abundant in the mutant-treated cells due to missing value imputation.

Supplementary Methods

General Synthesis Methods. Reagents and solvents were purchased from commercial suppliers (SigmaAldrich Co. LLC, Thermo Fisher Scientific Inc., Merck KGaA, TCI Europe GmbH, Fluorochem Ltd. and Alfa Aesar GmbH) and used without further purification. HPLC-grade solvents or anhydrous solvents (max. 0.01 % water content, stored over molecular sieve under an argon atmosphere, Sigma-Aldrich Co. LLC) were used for all reactions. All experiments were monitored by analytical thin layer chromatography (TLC). TLC was performed on pre-coated silica gel plates (60 F-254, 0.25 mm, Merck KGaA) with detection by UV (λ = 254 and/or 366 nm) and/or by colouration using a potassium permanganate (KMnO4) stain and subsequent heat treatment. Flash chromatography was performed on silica gel 60 (0.035 - 0.070 mm, mesh 60 Å, Merck KGaA) with the indicated eluent. 1 H, proton-decoupled 13C and protondecoupled 31P NMR spectra were recorded on a Bruker Avance III HD 300 (300 MHz), a Bruker Avance I 360 (360 MHz) or a Bruker Avance III HD (500 MHz) at 298 K. Chemical shifts are reported in delta (δ) units in parts per million (ppm) relative to distinguished solvent signals. The following abbreviations are used for the assignment of the signals: s - singlet, d - doublet, t - triplet, q - quartet, m - multiplet. Coupling constants J are given in Hertz [Hz]. HR-MS spectra were recorded in the ESI mode on a Thermo Scientific LTQ-FT Ultra (FT-ICR-MS) coupled with an UltiMate 3000 HPLC system (Thermo Fisher Scientific Inc.). Where explicitly indicated in the procedure, air and moisture-sensitive reactions were performed under inert conditions. In such cases, the glass apparatus was dried using a hot air pistol and the atmosphere was purged with argon. The reaction was then performed under positive pressure of argon. Dry solvents for use in reactions were purchased from Acros Organics or Sigma Aldrich and used without further purification. Dry deuterated solvents for the NMR measurements were purchased from Deutero. During workup and flash column chromatography, technical grade solvents were employed. All reagents were used as received from commercial suppliers (Sigma Aldrich, Alpha Aesar, Acros Organics, TCI) without further purification unless otherwise stated. Reaction progress was monitored using qualitative TLC on aluminum plates pre-coated with silica gel 60 (0.2mm thickness, F_{254} fluorescent indicator,

Merck). Visualization was achieved by UV light (λ = 254 nm and λ = 366 nm) or by treatment with a basic potassium permanganate ($KMnO_4$) stain followed by heating. Column chromatography was performed using silica gel 60 (230-400 Mesh, Acros) and the indicated solvent system(s). ^{1}H, ^{13}C and ^{31}PNMR spectra were recorded on a Bruker AVHD-300, AVHD-400, AVHD- 500 or AVII-500 (the latter equipped with a cryo probe) spectrometer at 298K. Chemical shifts (δ) are given in parts per million (ppm) and referenced to the residual solvent peak (^{1}H NMR: DMSO d6 δ = 2.50 ppm; ^{13}C NMR: DMSO d6 δ = 39.52 ppm, $CDCl_3$ δ = 77.16 ppm). In the case of ^{31}P NMR data, chemical shifts are reported without calibration relative to an internal standard as these spectra were measured solely for the purpose of assessing purity. Scalar coupling constants (J) are reported in Hertz (Hz), indicating the nature of the coupling nuclei as well as the number of bonds between them. ^{1}H NMR splitting patterns were designated as singlet (s), doublet (d), triplet (t), quartet (q) and combinations of these. Splitting patterns that could not be interpreted or easily visualized were designated as multiplet (m). Multiplets resulting from coincidentally equal coupling constants of magnetically inequivalent protons were marked as virtual (virt.). All ^{13}C and ^{31}P NMR and spectra were measured proton-decoupled. The assignment of signals, including ^{13}C nuclei numbers, was accomplished using two-dimensional NMR experiments (COSY, HSQC, HMBC) and doublets caused by the P-epimeric structure were averaged and reported as one. Only unambiguous signals were assigned. Spectra were processed and analyzed using the MestReNova software package from Mestrelab Research. The “Whittaker Smoother” baseline correction was applied. High-resolution mass spectra (HRMS) were measured using a Thermo Fisher Scientific Orbitrap XL spectrometer (electrospray ionisation in positive mode, ESI^+) coupled to an UltiMate 3000 UHPLC system (Thermo Fisher Scientific) in order to assess compound identity as well as purity. Relevant signals are reported as mass-to-charge ratios (m/z). Molecular ions are abbreviated as M.

***N*3-Propargyluridine.** To a solution of uridine (1.00 g, 4.09 mmol, 1.00 eq.) in a mixture of DMF (5 mL) and acetone (5 mL) was added finely ground K_2CO_3 (1.02 g, 7.37 mmol, 1.80 eq.) and the suspension was stirred at r.t. for 1h. Propargyl bromide (0.46 mL, 731 mg, 6.14 mmol,

1.50 eq.) was added dropwise at r.t. and the reaction mixture was stirred at 55 °C for additional 2h. Subsequently, the suspension was filtered and the solvents were evaporated under reduced pressure, repeatedly adding toluene to form a low-boiling azeotrope with DMF (3x). Purification of the crude product by column chromatography on silica (CH_2Cl_2/MeOH = 95/5 → 90/10) afforded uridine derivative **2** as a white foam (1.04 g, 4.09 mmol, 90%). ^{1}H NMR (300 MHz, DMSO-d$_6$) : δ (ppm) = 8.00 (d, $^3J_{H,H}$ = 8.2 Hz, 1H), 5.87 - 5.80 (m, 2H), 5.42 (d, $^3J_{H,H}$ = 5.7 Hz, 1H), 5.15-5.07 (m, 2H), 4.52 (d, $^4J_{H,H}$ = 2.3 Hz, 2H), 4.08-4.01 (m, 1H), 3.97 (*virt.* q, $^3J_{H,H}$ = 4.8 Hz, 1H), 3.90-3.84 (m, 1H), 3.69-3.51 (m, 2H), 3.11 (*virt.* t, $^4J_{H,H}$ = 2.3Hz, 1H, H). HRMS (ESI+): m/z calcd. for $C_{12}H_{15}N_2O_6^+$ ([M+H]$^+$): 283.0925, found: 283.0921; calcd. for $C_{12}H_{14}N_2O_6Na^+$ ([M+Na]$^+$): 305.0744, found: 305.0740.

2′,3′-*O*-Isopropylidene-*N*3-propargyluridine. To a solution of alkylated uridine **2** (800 mg, 2.83 mmol, 1.00 eq.) in acetone (28 mL) was added 2,2-dimethoxypropane (3.49 mL, 2.95 g, 28.3 mmol, 10.0 eq.) as well as *p*toluenesulphonic acid hydrate (53.9 mg, 238 µmol, 10 mol-%). The reaction mixture was stirred at r.t. for 2h and subsequently partitioned between sat. aq. $NaHCO_3$ and EtOAc. The aqueous phase was extracted with EtOAc two additional times. The combined organic phases were washed with sat. aq. NaCl (1x), dried over $MgSO_4$, filtered and concentrated under reduced pressure. Purification of the crude product by column chromatography on silica (CH_2Cl_2/MeOH = 95/5) afforded protected uridine derivative **3** as a white foam (534 mg, 1.66 mmol, 58%). ^{1}H NMR (400 MHz, DMSO-d$_6$): δ (ppm) = 7.89 (d, $^3J_{H,H}$ = 8.1 Hz, 1H), 5.88 (d, $^3J_{H,H}$ = 2.6Hz, 1H), 5.82 (d, $^3J_{H,H}$ = 8.1 Hz, 1H), 5.12 (*virt.* t, $^3J_{H,H}$ = 5.2 Hz,1H), 4.90 (dd, $^3J_{H,H}$ = 6.3, 2.6 Hz, 1H), 4.76 (dd, $^3J_{H,H}$ = 6.3, 3.3 Hz, 1H), 4.51 (d, $^4J_{H,H}$ = 2.4 Hz, 2H), 4.13 (*virt.* q, $^3J_{H,H}$ = 4.1 Hz, 1H), 3.65-3.52 (m, 2H), 3.13 (*virt.* t, $^4J_{H,H}$ = 2.4 Hz, 1H), 1.49 (s, 3H), 1.29 (s, 3H). HRMS (ESI$^+$): *m/z* calcd. for $C_{15}H_{18}N_2O_6Na^+$ ([M+Na]$^+$): 345.1057, found: 345.1056.

2′,3′-*O*-Isopropylidene-*N*3-propargyluridine-5′-*O*-[phenyl(benzyloxy-l-alaninyl)]-phosphate. L-Alanine benzyl ester *p*-toluenesulphonate (1.18 g, 3.35 mmol, 1.00 eq.) was added to a dried, argon-purged flask and dissolved in dry CH_2Cl_2 (17 mL). Phenyl dichlorophosphate (0.50 mL, 706 mg, 3.35 mmol, 1.00 eq.) was added and the solution was cooled down to −78 °C using a dry ice/acetone bath. NEt_3 (0.93 mL, 677 mg, 6.69 mmol, 2.00 eq.) was added dropwise. Thereafter, the reaction mixture was warmed up to r.t. and stirred for additional 1h. The volatiles were evaporated under reduced pressure and the residue was triturated with THF. The suspension was filtered and the filtrate was concentrated under reduced pressure to afford **4** as a colourless oil. The crude product was used without further purification. Protected uridine derivative **3** (332 mg, 1.03 mmol, 1.00 eq.) was added to a dried, argon purged flask and dissolved in dry THF (10 mL). A solution of *tert*-butylmagnesium chloride (1 M in THF, 2.06 mL, 2.06 mmol, 2.00 eq.) was added dropwise and the solution was stirred for 30min at r.t. A solution of crude compound **4** (910 mg, approx. 2.57 mmol, approx. 2.50 eq.) in THF (10mL) was then added dropwise and the reaction mixture was stirred at r.t. for additional 1.5 h. Subsequently, the mixture was partitioned between sat. aq. NH_4Cl and EtOAc. The aqueous phase was extracted with EtOAc two additional times. The combined organic phases were dried over $MgSO_4$, filtered and concentrated under reduced pressure. Purification of the crude compound by column chromatography on silica (pentane/EtOAc = 30/70) afforded protected phosphoramidate **5** as a white foam (approx. 1/1 mixture of diastereomers, 569mg, 89 wt-% with EtOAc as main impurity, 792 µmol, 77%). ^{1}H NMR (500 MHz, DMSO-d$_6$): δ (ppm) = 7.74 (d, $^3J_{H,H}$ = 8.1 Hz, 1H), 7.73 (d, J = 8.1 Hz, 1H), 7.38-7.29 (m, 14H), 7.20-7.11 (m, 6H), 6.17-6.09 (m, 2H), 5.86 (d, $^3J_{H,H}$ = 2.4 Hz, 1H), 5.85 (d, $^3J_{H,H}$ = 2.4 Hz, 1H), 5.79 (d, $^3J_{H,H}$ = 8.1 Hz, 1H), 5.75 (d, $^3J_{H,H}$ = 8.1 Hz, 1H, H), 5.13-5.05 (m, 4H), 4.96 (dd, $^3J_{H,H}$ = 6.4, 2.4 Hz, 1H), 4.83 (dd, $^3J_{H,H}$ = 6.4,2.4 Hz, 1H), 4.79 (dd, $^3J_{H,H}$ = 6.4,3.5 Hz, 1H), 4.75 (dd, $^3J_{H,H}$ = 6.4,3.5 Hz, 1H), 4.49 (d, $^4J_{H,H}$ = 2.5 Hz, 2H), 4.48 (d, $^4J_{H,H}$ = 2.5 Hz, 2H), 4.31-4.07 (m, 6H), 3.97-3.87 (m, 2H), 3.13-3.10 (m, 2H), 1.49 (s, 6H), 1.29 (s, 6H), 1.27 (d, $^3J_{H,H}$ = 7.2 Hz, 3H), 1.24 (d, $^3J_{H,H}$ = 7.2 Hz, 3H). HRMS (ESI$^+$): *m*/*z* calcd. for $C_{31}H_{35}N_3O_{10}P^+$ ([M+H]$^+$):

640.2055, found: 640.2057; calcd. for $C_{31}H_{34}N_3O_{10}PNa^+$ ($[M+Na]^+$): 662.1874, found: 662.1869.

***N*3-propargyluridine-5′-*O*-[phenyl(benzyloxy-l-alaninyl)] phosphate (pro-N3pU).** Protected phosphoramidate **5** (569 mg, 89 wt-%, 792µ mol, 1.00 eq.) was dissolved in 90% v/v aq. TFA (10mL) and stirred at r.t. for 3h. Thereafter, volatiles were repeatedly co-evaporated with MeOH under reduced pressure. Purification of the crude compound by column chromatography on silica (CH_2Cl_2/MeOH = 97/3 → 95/5) afforded phosphoramidate **pro-N3pU** as a white foam (approx. 1/1 mixture of diastereomers, 357 mg, 595 µmol, 75 %). ^{1}H NMR (500 MHz, DMSO-d_6): δ (ppm) = 7.68 (d, $^3J_{H,H}$ = 8.1 Hz, 1H), 7.66 (d, $^3J_{H,H}$ = 8.1 Hz, 1H), 7.38-7.29 (m, 14H), 7.22-7.14 (m, 6H), 6.19-6.11 (m, 2H), 5.84-5.80 (m, 2H), 5.75 (d, $^3J_{H,H}$ = 8.1 Hz, 1H), 5.70 (d, $^3J_{H,H}$ = 8.1 Hz, 1H), 5.56-5.51 (m, 2H), 5.34 (d, J = 5.4 Hz, 1H), 5.32 (d, J = 5.4 Hz, 1H), 5.13-5.05 (m, 4H), 4.57-4.46 (m, 4H), 4.25-3.85 (m, 12H), 3.14-3.11 (m, 2H), 1.26 (d, $^3J_{H,H}$ = 7.0 Hz, 3H), 1.24 (d, $^3J_{H,H}$ = 7.0 Hz, 3H). ^{13}C{^{1}H} NMR (126 MHz, DMSO-d6): δ (ppm) = 173.1, 160.9, 150.4, 139.6, 135.9, 129.7, 128.3, 127.9, 124.7, 120.2, 101.1, 101.1, 89.2, 82.3, 78.9, 73.2, 72.9, 69.5, 66.1, 65.7, 49.9, 29.9, 19.7. ^{31}P{^{1}H} NMR (162 MHz, DMSO-d6): δ (ppm) = 3.81, 3.80. HRMS (ESI$^+$): m/z calcd. for $C_{28}H_{31}N_3O_{10}P^+$ ($[M+H]^+$): 600.1742, found: 600.1746; calcd. for $C_{28}H_{30}N_3O_{10}PNa^+$ ($[M+Na]^+$): 622.1561, found: 622.1556. TLC: R_f = 0.33 ($CH2Cl_2$/MeOH = 95/5) [UV/$KMnO_4$].

NMR Spectra

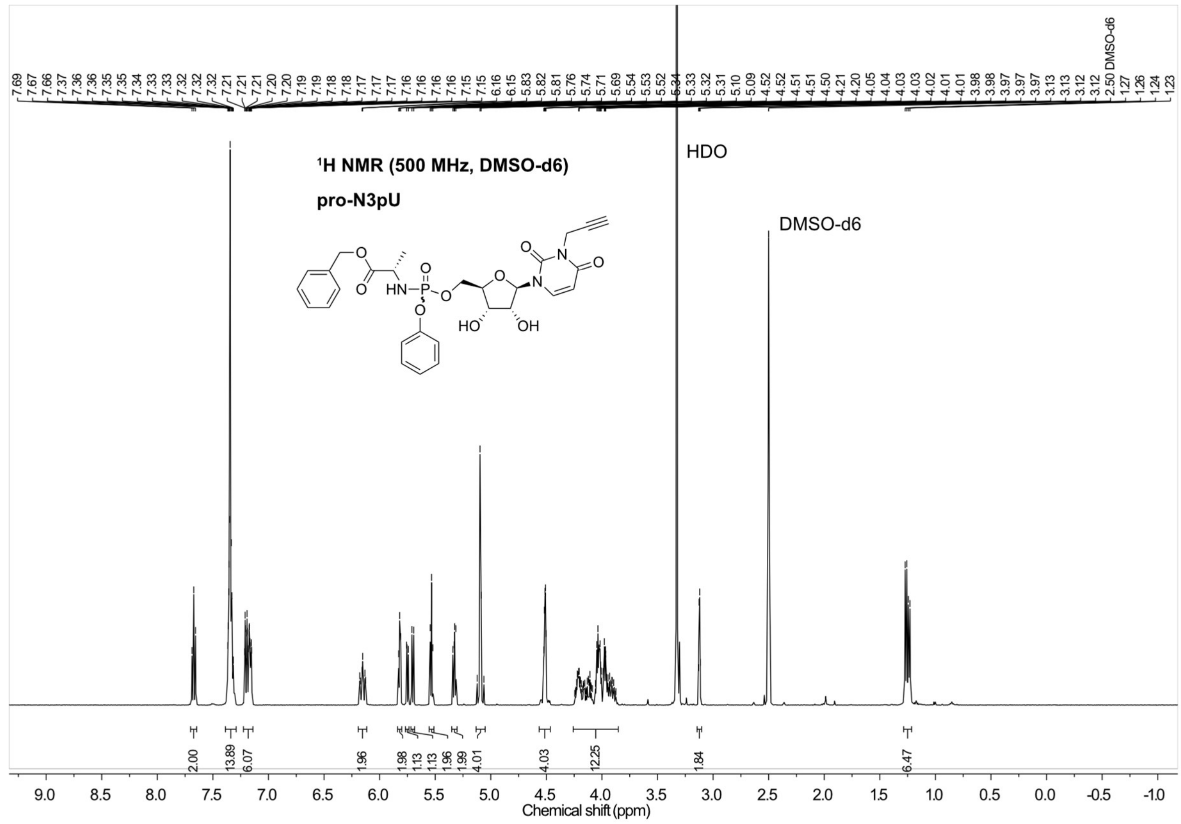

^1H NMR (500 MHz, DMSO-d6)
pro-N3pU
HDO
DMSO-d6
Chemical shift (ppm)

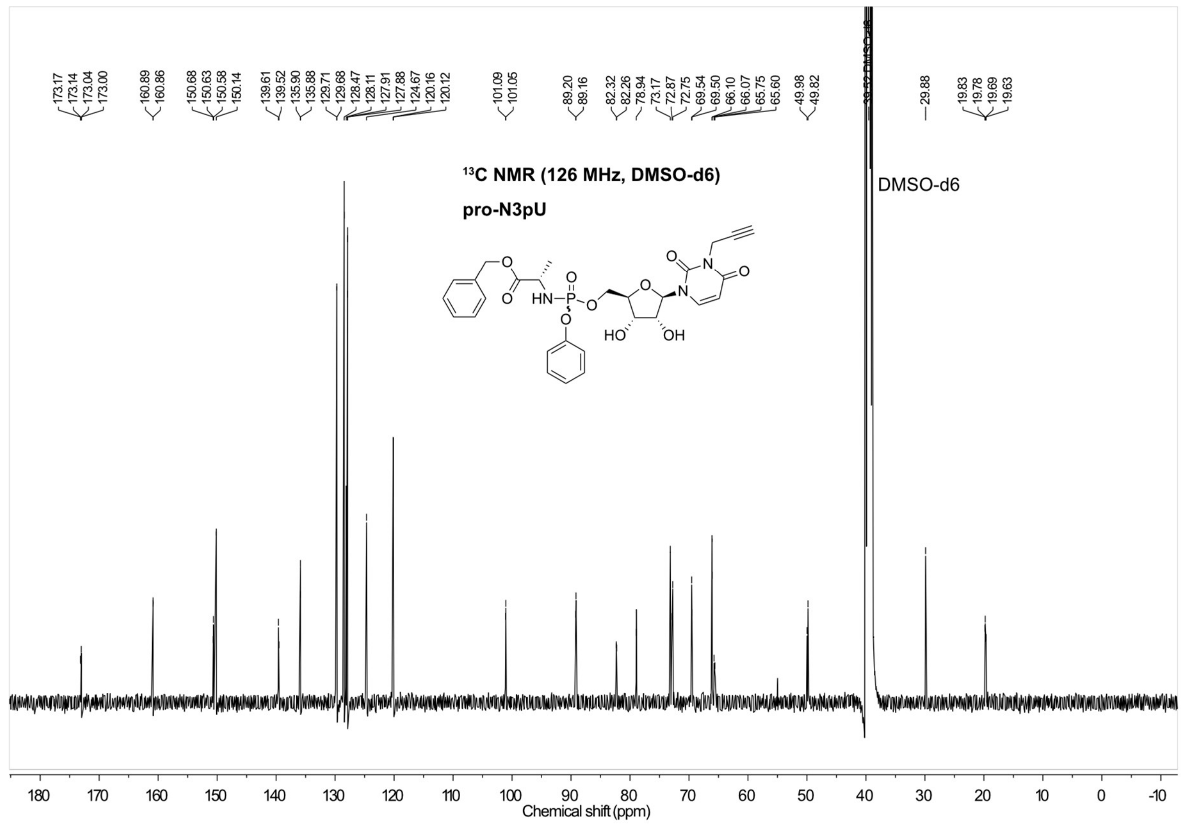

^{13}C NMR (126 MHz, DMSO-d6)
pro-N3pU
DMSO-d6
Chemical shift (ppm)

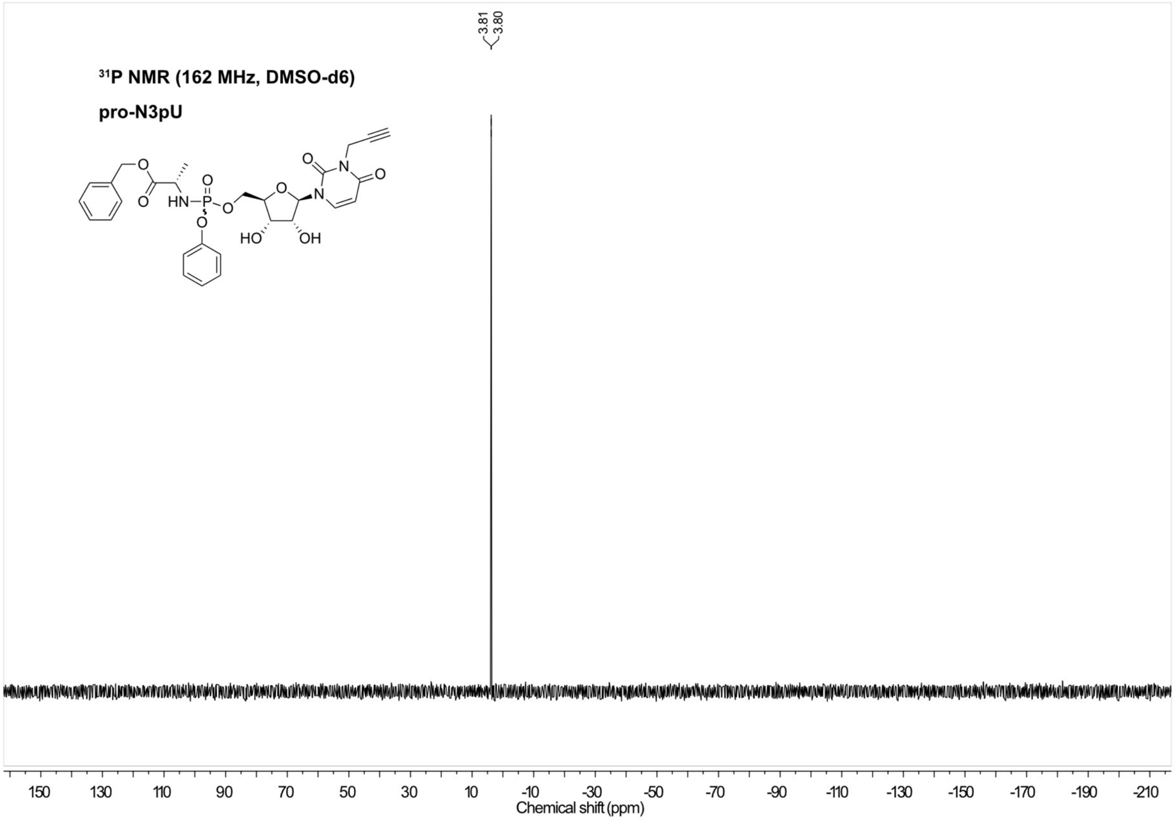

3.81
3.80
31P NMR (162 MHz, DMSO-d6)
pro-N3pU
150 130 110 90 70 50 30 10 -10 -30 -50 -70 -90 -110 -130 -150 -170 -190 -210
Chemical shift (ppm)
